# Correlation of hyponatremia, leukocytosis, hypomagnesemia and fever after sah with delayed cerebral ischemia and poor outcome

**DOI:** 10.1186/2197-425X-3-S1-A783

**Published:** 2015-10-01

**Authors:** V Vrsajkov, N Mančić, M Galešev, D Glišić

**Affiliations:** Clinical Centre of Vojvodina, Emergency Centre, Novi Sad, Serbia; Clinical Centre of Vojvodina, Clinic of Anesthesia and Intensive Therapy, Novi Sad, Serbia

## Introduction

Early identification of patients at an increased risk for delayed cerebral ischemia (DCI) and poor outcome could allow more aggressive therapy and influence better outcome.

## Objectives

The aim of this study was to determine a predictive association of hyponatremia, hypomagnesemia, fever and leukocytosis with DCI and poor outcome.

## Methods

We prospective enrolled 68 patients with SAH treated from March 2011 to May 2013. Serum levels of sodium, magnesium and leukocyte count were determined at least once a day during the first 10 days after SAH. All patients underwent noncontrast CT scan 9 ± 2 days after SAH. DCI was defined as one or more of the next parameters: a new focal neurological deficit, decline for 2 or more points on the mGCS scale or a new hypodensity on CT scan. The outcome was assessed after 6 months using the eGOS scale.

## Results

48% of the patients recruited had delayed cerebral ischemia. Logistic regression model showed significant impact of hyponatremia (p=0,036 OR=4.08 95% CI = 1.09 - 15.26) on DCI and poor outcome (p=0,034 OR=5.11 95% CI = 1.13 - 23.14). We obtained strong corelation of leukocytosis (p = 0.013) with DCI and poor outcome (p=0,016). Association of non infectious fever and hypomagnesemia with DCI existed but wasn´t significant enough.

## Conclusions

Our results results confirmed association of hyponatremia and leukocytosis with greater risk of developing DCI and poor clinical outcome.
